# MMseqs2 desktop and local web server app for fast, interactive sequence searches

**DOI:** 10.1093/bioinformatics/bty1057

**Published:** 2019-01-07

**Authors:** Milot Mirdita, Martin Steinegger, Johannes Söding

**Affiliations:** bty1057-aff1 Quantitative and Computational Biology, Max Planck Institute for Biophysical Chemistry, Göttingen, Germany; bty1057-aff2 Department of Chemistry, Seoul National University, Seoul, Korea

## Abstract

**Summary:**

The MMseqs2 desktop and web server app facilitates interactive sequence searches through custom protein sequence and profile databases on personal workstations. By eliminating MMseqs2’s runtime overhead, we reduced response times to a few seconds at sensitivities close to BLAST.

**Availability and implementation:**

The app is easy to install for non-experts. GPLv3-licensed code, pre-built desktop app packages for Windows, MacOS and Linux, Docker images for the web server application and a demo web server are available at https://search.mmseqs.com.

**Supplementary information:**

Supplementary data are available at *Bioinformatics* online.

## 1 Introduction

The most popular sequence similarity search tool, BLAST (Altschul *et al.*, 1990, 1997), has garnered ∼7000 citations per year during the last 5 years, attesting to the unremitting importance of sequence searches for biology. This popularity may be largely owed to the excellent web services with short response times despite fast-growing databases provided by the NCBI/NIH, which requires a huge compute infrastructure. The distributed approach of running searches *locally* on personal computers or IT platforms of companies and research groups allows for custom databases, high availability and protects sensitive data. But web server applications for local homology searches are slow as they mostly rely on BLAST (e.g. Deng *et al.*, 2007; Priyam *et al.*, 2015). Here, we present an application software to search with protein and nucleotide sequences through custom protein sequence and profile databases using MMseqs2 (Steinegger and Söding, 2017), achieving response times of seconds instead of minutes at a similar sensitivity as BLAST.

## 2 Materials and methods

### 2.1 Reduced runtime overhead

MMseqs2 owes its sensitivity and speed mainly to its pre-filtering stage, which rejects ∼99.99% of sequences. The pre-filter uses a reverse *k*-mer index table for the target database and also requires matrices with similarity scores between 2-mers and between 3-mers to generate the lists of similar 7-mers (Steinegger and Söding, 2017). Reading in the index table and computing these matrices on-the-fly takes ∼0.5 min of runtime overhead for each search. We reduced this to 0.05 s by (1) writing the index table, the matrices and other pre-computable data into a file if it does not yet exist, memory mapping the file to take advantage of the system page cache (for detailed memory requirements see Supplementary Materials) and (3) optimizing I/O operations.

### 2.2 Optimized sequence-to-profile search mode

The index table for profile databases stores, for each position in a profile, all *k*-mers with a profile similarity score above a threshold set by –s. The number of similar *k*-mers grows exponentially with *k*. To save memory, we chose a short *k *=* *5 as default for this mode. We also added to Mmseqs2 utilities for creating profiles from multiple sequence alignments (MSAs) and converting between profile formats.

### 2.3 Desktop and web server app

Based on the same code base, the application can be either deployed through Docker containers to be accessed through web browsers or packaged as a desktop GUI application with the Electron framework (electronjs.org). In either case, the backend part of the application provides a RESTful API and worker scheduling. The server supports protein, translated nucleotide and nucleotide sequence searches and iterative and reverse profile searches.

**Fig. 1.**
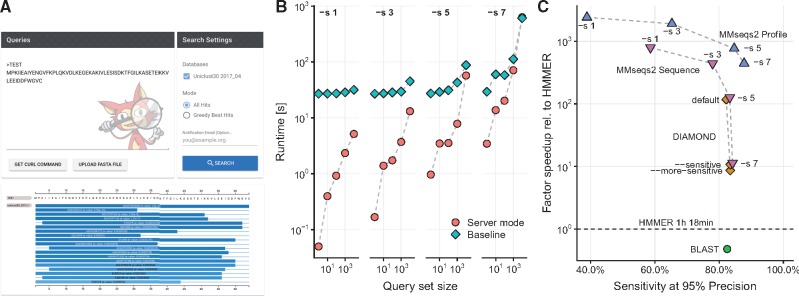
(**A**) Screenshots of the search interface and result visualization. (**B**) Runtime of searches with the baseline MMseqs2 (square) and the new server mode (circle) at four sensitivity settings (-s). (**C**) Domain annotation: Speedup versus sensitivity at 95% precision for MMseqs2 (triangle: sequence-profile search, upside-down triangle: sequence–sequence search; sensitivity settings: -s 1, 3, 5, 7), DIAMOND (square; *default*, –sensitive, –more-sensitive) and BLAST (circle). HMMER3 matches to Pfam domains are used as ground truth. The speed-ups exclude the times to format the databases

The application takes a list of either protein or nucleotide sequences in FASTA/FASTQ format as query input. To generate a target search database, the application takes a FASTA/FASTQ file for protein sequence searches or a STOCKHOLM MSA file for protein profile searches. Search results are shown with a customized feature-viewer (github.com/calipho-sib/feature-viewer) (Fig. 1A) and can be downloaded in tabular BLAST format.

## 3 Results

Figure 1B demonstrates the reduction of runtime overhead by comparing the runtimes of the Mmseqs2 version without (‘baseline’) to the new version with pre-computations and memory mapping (‘server mode’). Runtimes refer to searches with amino acid query sets of 1, 10, 100, 1000 and 10 000 sequences of average length 350 (sampled from the Uniclust30 database) through the Uniclust30 2017_10 database (Mirdita *et al.*, 2017) with 13.5 million sequences, measured on a server with 2 Intel Xeon E5-2680 v4 CPUs with 14 cores each. The index table and matrix pre-computation (∼3 min 40 s) is not included in the runtimes.

To test the quality and speed of annotating Pfam domains on genes assembled from metagenomics data, we built a test set by sampling 100 000 full-length sequences longer than 150 residues from our Marine Eukaryotic Reference Catalogue (Steinegger *et al.*, 2018), clustering this set to 30% maximum pairwise sequence identity with MMseqs2 and sampling 10 000 sequences from the redundancy-reduced set. We annotated these sequences with PfamA 31.0 domains (Finn *et al.*, 2014) using HMMER3 (Finn *et al.*, 2011).

We then compared how well the sequence-sequence searches of MMseqs2, BLAST and DIAMOND (Buchfink *et al.*, 2015) and the sequence-to-profile searches of MMseqs2 could find the correct domain annotations. For the sequence-sequence search methods, we built a database from all sequences in PfamA.full MSAs and reported as E-value of a Pfam domain the E-value for the best-matching sequence from its MSA. We defined a search as true positive (TP) if the top match was annotated by HMMER3 with an E-value better than 10^−3^ and as false positive (FP) if the top match was not annotated with an HMMER3 E-value below 1. All other searches were considered ambiguous and ignored. For each method, we determined the E-value at which the precision TP/(TP+FP) is 95% and measured the sensitivity at that E-value.

As Figure 1C shows, MMseqs2 sequence-to-profile searches are ∼30 times faster than sequence-sequence searches with DIAMOND, MMseqs2 and BLAST and ∼300 times faster than HMMER3. MMseqs2 sequence-to-profile searches reach 87% relative sensitivity at 95% precision, making them an attractive alternative to HMMER3 when speed is critical.

## 4 Conclusion

The desktop and web server app for MMseqs2 performs fast sequence searches at unprecedented speed-to-sensitivity trade-off on local computers. Thousand queries take only a minute to search through fifteen million sequences of the Uniclust30 database, much faster than NCBI’s BLAST website. We hope the MMseqs2 app will also empower users unfamiliar with command line interfaces to perform fast and sensitive searches with their own sequence and profile databases.

## Supplementary Material

bty1057_Supplementary_Data

## References

[bty1057-B1] Altschul S.F. et al (1990) Basic local alignment search tool. J. Mol. Biol., 215, 403–410. 2231712 10.1016/S0022-2836(05)80360-2

[bty1057-B2] Altschul S.F. et al (1997) Gapped BLAST and PSI-BLAST: a new generation of protein database search programs. Nucleic Acids Res., 25, 3389–3402. 9254694 10.1093/nar/25.17.3389 PMC146917

[bty1057-B3] Buchfink B. et al (2015) Fast and sensitive protein alignment using DIAMOND. Nat. Methods, 12, 59–60. 25402007 10.1038/nmeth.3176

[bty1057-B4] Deng W. et al (2007) Viroblast: a stand-alone blast web server for flexible queries of multiple databases and user’s datasets. Bioinformatics, 23, 2334–2336. 17586542 10.1093/bioinformatics/btm331

[bty1057-B5] Finn R.D. et al (2011) HMMER web server: interactive sequence similarity searching. Nucleic Acids Res., 39, W29–W37. 21593126 10.1093/nar/gkr367 PMC3125773

[bty1057-B6] Finn R.D. et al (2014) Pfam: the protein families database. Nucleic Acids Res., 42, D222–D230. 24288371 10.1093/nar/gkt1223 PMC3965110

[bty1057-B7] Mirdita M. et al (2017) Uniclust databases of clustered and deeply annotated protein sequences and alignments. Nucleic Acids Res., 45, D170–D176. 27899574 10.1093/nar/gkw1081 PMC5614098

[bty1057-B8] Priyam A. et al (2015) Sequenceserver: a modern graphical user interface for custom BLAST databases. bioRxiv, 033142. 10.1093/molbev/msz185 PMC6878946 31411700

[bty1057-B9] Steinegger M., Söding J. (2017) MMseqs2 enables sensitive protein sequence searching for the analysis of massive data sets. Nat. Biotechnol., 35, 1026–1028. 29035372 10.1038/nbt.3988

[bty1057-B10] Steinegger M. et al (2018) Protein-level assembly increases protein sequence recovery from metagenomic samples manyfold. bioRxiv, 386110. 10.1038/s41592-019-0437-4 31235882

