# Supplementary material for: MMseqs2 desktop and local web server app for fast, interactive sequence searches
Source: Bioinformatics. 2019 Jan 7;35(16):2856–8. doi: 10.1093/bioinformatics/bty1057 (PMC6691333; doi:10.1093/bioinformatics/bty1057)
Supplement: bty1057_Supplementary_Data [file bty1057_supplementary_data.pdf]

Supplementary Material for MMseqs2 desktop and local web server app for fast, interactive sequence searches

Mirdita M.,[1] Steinegger M.,[1,2] and Söding J.[1]

[1]*Quantitative and Computational Biology Group, Max Planck Institute for Biophysical Chemistry, Göttingen, Germany*
[2]*Department of Chemistry, Seoul National University, Seoul, Korea*

I. MEMORY REQUIREMENTS

The MMseqs2 web server keeps the precomputed index fully in main memory using memory mapping with the `mmap` system call.

For optimal speed, the whole precomputed index file has to reside in the operating system's page cache.

The precomputed index consists predominantly of two parts: (1) the k-mer lookup table and (2) the residues. The memory consumption grows linearly with the number of residues in the database.

A. Sequence-sequence search requirements

The following formula can be used to estimate the size M of the precomputed index file in the case of a sequence-sequence search.

$$\begin{aligned} M &\approx 8\,\mathrm{b} \times N \times L \\ &+ 8\,\mathrm{b} \times a^k \\ &+ 32\,\mathrm{b} \times N \end{aligned}$$

Where N is the database size, L is the average sequence length, a the alphabet size (typically 20, with the unknown residue X excluded) and k the k-mer size.

The following table shows memory requirements for a few example databases of different sizes:

Name	Release	Entries	Size $k=6$	Size $k=7$
SwissProt	2018_08	558125	2.3GB	12GB
Uniclust30	2018_08	30M	56GB	64GB
Uniclust50	2018_08	45M	96GB	105GB
Uniclust90	2018_08	120M	295GB	304GB

TABLE I. Memory requirements for typical sequence search databases.

B. Sequence-profile search requirements

The target profile search keeps all similar k-mers for each profile in memory. The memory consumption M is dominated by the average k-mer list length (K_{avg}) per profile column. K_{avg} depends on the chosen sensitivity setting, higher sensitivity results in longer k-mer lists.

$$M \approx 6\,\mathrm{b} \times N \times L_p \times K_{\mathrm{avg}}$$

Where N is the database size, L_p is the average profile column length.

The following table shows memory requirements and typical K_{avg} values for the Pfam-A profile database at different sensitivity settings:

Sensitivity	$k=5$	K_{avg}	$k=6$	K_{avg}
$s=1$	123MB	4.7	1.1GB	19.2
$s=3$	633MB	26	5.0GB	190
$s=5$	5.3GB	231	25GB	1070
$s=7$	26GB	1401	119G	5279

TABLE II. Memory requirements for Pfam-A 31.0 profile search databases (16479 profiles, 4006517 total residues in consensus sequences).

II. SOFTWARE VERSIONS

Name	Version
MMseqs2	Git: 8a8520c
blastp	2.6.0+
hmmer	3.1b2
diamond	0.9.19

TABLE III. Software versions used in this manuscript.
